# Brain Mechanisms Underlying Visuo-Orthographic Deficits in Children With Developmental Dyslexia

**DOI:** 10.3389/fnhum.2018.00490

**Published:** 2018-12-06

**Authors:** Fan Cao, Xin Yan, Gregory J. Spray, Yanni Liu, Yuan Deng

**Affiliations:** ^1^Department of Psychology, Sun Yat-sen University, Guangzhou, China; ^2^Department of Communicative Sciences and Disorders, Michigan State University, East Lansing, MI, United States; ^3^School of Humanities and Social Science, The Chinese University of Hong Kong, Shenzhen, Shenzhen, China; ^4^Department of Psychiatry, University of Michigan, Ann Arbor, MI, United States; ^5^Institute of Psychology, Chinese Academy of Sciences, Beijing, China

**Keywords:** dyslexia, fMRI, orthographic deficit, visual deficit, PPI

## Abstract

Multiple hypotheses have been proposed to explain the reading difficulty caused by developmental dyslexia (DD). The current study examined visuo-orthographic processing in children with dyslexia to determine whether orthographic deficits are explainable based solely on visual deficits. To identify orthographic-specific, visual perception-specific, and overlapping deficits, we included two tasks (lexical and perceptual) in three Chinese subject groups: children with DD, age-matched controls (AC), and reading matched controls (RC) using functional magnetic resonance imaging (fMRI). We found that the left precuneus showed decreased activation across both tasks for the DD group compared to the two control groups, thus reflecting visual processing deficits in children with DD, which also affects orthographic processing. Furthermore, we found that the functional connectivity between left middle occipital gyrus (LMOG) and left inferior frontal gyrus (IFG) was decreased in the DD group compared to AC and RC for only the lexical task. This suggests a weaker association between orthography and phonology for children with DD. In addition, the children with DD showed decreased functional connectivity between the LMOG and right parahippocampal gyrus for only the visual perceptual task, thereby indicating a weaker association between visual regions for DD during visual symbol processing. Taken together, our findings suggest that the observed orthographic processing deficit in DD might be driven by both a basic visual deficit, and a linguistic deficit.

## Introduction

Developmental dyslexia (DD) is characterized as a specific and significant impairment in reading ability, which cannot be explained by deficits in general intelligence, motivation, or educational opportunity (Critchley, 1970). The phonological deficit hypothesis is one of the most commonly used theories to explain the etiology of dyslexia, and speculates that underspecified phoneme representations or the unsuccessful retrieval of phoneme representations are the core causes of reading difficulties in readers with dyslexia (Snowling, 1980; Muter et al., 1998; Boets et al., 2013). In addition to behavioral studies, evidence from neuroimaging studies have also documented support for the phonological deficit hypothesis. For example, reduced brain activation has been reported among left temporo-parietal areas during phonological processing in both the visual (Paulesu et al., 2001; Schulz et al., 2009; Van der Mark et al., 2009; Tanaka et al., 2011) and auditory modalities (Eden et al., 2004; Dufor et al., 2007; Kast et al., 2011). The left temporo-parietal region has been associated with phonological representation and the conversion between orthography and phonology (McCrory et al., 2000; Blau et al., 2010; Kast et al., 2011; Pecini et al., 2011; Paulesu et al., 2014). Reduced brain activation has also been found in the left inferior frontal gyrus (IFG) (Hoeft et al., 2006; Richlan et al., 2011; Cao et al., 2017), which has been associated with phonological segmentation and manipulation during phonological awareness tasks (Pugh et al., 1996; Fiez, 1997; Tan et al., 2001).

Although an abundance of evidence supports the phonological deficit hypothesis, the orthographic deficits observed in readers with dyslexia cannot be overlooked. Indeed, reading is a complex process that involves extensive orthographic analysis of letters, letter strings, and word recognition. People with dyslexia have shown to exhibit difficulties identifying letters within letter strings (Bouma and Legein, 1977; Geiger and Lettvin, 1987), selecting the correct spelling of a target word presented with homophones (Coltheart et al., 1983), and identifying words with similar orthography (Hawelka et al., 2006; Ziegler et al., 2010). Researchers have argued that orthographic deficits may be due to limited exposure and experience with the writing system or inadequate instruction, rather than dyslexia (Mol and Bus, 2011; Huettig et al., 2018). However, compared to reading-matched children, children with DD have been reported to show deficient orthographic processing (Suarez-Coalla et al., 2014), thereby suggesting that orthographic deficits are not purely due to developmental delay.

In opaque and coarse-grained orthographies, such as Hebrew and Chinese, orthographic skills seem to play an even more prominent role in reading acquisition. This is because there are no obvious rules to map orthography to phonology in these languages, and there are many homophones. Therefore, reading these orthographies relies on direct mapping from orthography to semantics to a greater degree, and accurate recognition of orthography is critical for successful reading acquisition. Moreover, Chinese characters consist of strokes in a two-dimensional square; therefore, the complex visual–spatial configurations increase the demand of visual–spatial analysis in reading (Cao et al., 2013). A number of previous studies have reported that orthographic processing skills play a more important role than phonological skills in Chinese reading development (McBride-Chang et al., 2005; Chung et al., 2012; Li et al., 2012; Zhang et al., 2012).

Researchers have found that DD is associated with an impairment in visual attention and visual–spatial analysis (Vidyasagar and Pammer, 2010; Collis et al., 2013), which might help explain an orthographic deficit. These impaired visual processes may be due, in part, to an abnormality within the magnocellular system, which is sensitive to moving stimuli and is involved in visual motion detection. Therefore, the magnocellular system plays an important role in identifying blurred and/or moving letters during reading (Stein and Walsh, 1997). A number of studies have reported anomalous function of the magnocellular system amongst individuals with DD (Meng et al., 2011; Franceschini et al., 2013; Qian and Bi, 2014). For example, Meng et al. (2011) reported that children with DD had a higher threshold for detecting coherent motion than controls, which also predicted overall performance on a Chinese orthographic judgment task. Based on the aforementioned studies, it appears that visual deficits might underlie orthographic deficits observed in DD.

Neurologically speaking, readers with dyslexia exhibit abnormal brain activation during visual and orthographic processing tasks. For example, it has been reported that, compared to controls, people with dyslexia exhibit decreased activation in the middle occipital gyrus (MOG) during visual-perception tasks such as number identification (Boros et al., 2016), symbol detection (Boros et al., 2016), and arrow shape judgment (Zhang et al., 2013). Interestingly, decreased activation in the MOG has also been reported for orthographic tasks that involve pseudoword reading (Shaywitz et al., 2002; Van der Mark et al., 2009; Dehaene et al., 2010; Boros et al., 2016), lexical decision making (Siok et al., 2004), font judgment (Siok et al., 2008), letter matching (Temple et al., 2001), and letter identification (Boros et al., 2016). In summary, the reduced activation in the MOG during visual and orthographic processing tasks in people with DD suggests deficits in visual processing.

Visual–spatial processing has been associated with the posterior parietal cortex (PPC), including the inferior parietal lobule (IPL), superior parietal lobule (SPL), and precuneus (Fiez et al., 1995; Mangun et al., 1998; Shen et al., 1999). Interestingly, people with dyslexia have been reported to exhibit reduced activation among these brain regions, which support visual–spatial processing. Previous studies have found that stimuli placing a greater tax on visual attention elicit greater activation within the left precuneus/superior parietal lobule in control children; however, children with DD do not exhibit this same increase in activation at this region (Peyrin et al., 2011), which suggests a deficit in the visual–spatial processing of visually complex stimuli. This same region has also been reported to play an important role in visuo-orthographic processing during Chinese visual word recognition, and show a developmental increase with age (Cao et al., 2009). Furthermore, studies have shown that children with dyslexia exhibit decreased brain activation in the SPL during tasks involving character size judgment compared to control children (Siok et al., 2009). More recently, it was reported that adults with dyslexia exhibit decreased activation in the left SPL, left precuneus, and the bilateral IPL compared to control subjects while performing a letter string identification task, relative to perceptual analysis, (Reilhac et al., 2013). Taken together, reduced activation in the PPC has been observed during both visual-perceptual and visual-orthographic tasks in people with DD.

Currently, the relationship between the visual deficit and orthographic deficit experienced by readers with DD has yet to be explored completely. Namely, is the orthographic deficit caused solely by visual deficits, or is there a language specific deficit that is not explained by the visual deficit? One way to answer this question is to directly compare the two deficits in a single study. Very few studies have addressed this issue, and existing studies have reported different findings. Temple et al. (2001) found that children with DD exhibited decreased activation in the bilateral occipital-parietal region (including the bilateral MOG, right precuneus, and left cingulate) compared to controls during a letter-matching task in comparison to a line-matching task. The authors suggested that the reduced activation in the occipital-parietal region was language specific; however, poorer performance on the letter-matching task may have been due to greater complexity among the stimuli. More recently, Boros et al. (2016) reported that the processing of digits, letters, and symbol strings was associated with reduced activation in the left MOG and the left visual word form area. These findings suggest that a task-universal neural deficit in visual processing exists among children with DD; however, the same brain region may be connected with different regions to conduct different neural calculations (e.g., Menon and Uddin, 2010). Therefore, it is necessary to examine functional connectivity to determine whether visual and orthographic deficits have the same brain mechanisms in DD.

In the current study, we investigated the brain mechanisms underlying visual processing and orthographic processing in children with DD compared to age-matched controls (AC) and reading-matched controls (RC). We examined both brain activation and functional connectivity to determine similarities and differences between visual deficits and orthographic deficits in children with DD. The current study is unique in that it utilizes two control groups to thoroughly examine whether visuo-orthographic deficits observed in DD are due to a delay in maturation or dyslexia.

## Materials and Methods

### Participants

A total of fifty-eight Chinese children were recruited from 8 public elementary schools in Beijing. Twenty-three fifth-grade children were defined as individuals with DD (mean age = 11.11, range: 10.11–12; 17 males); 19 fifth-grade children served as AC (mean age = 11.03, range: 10.11–12.03; 10 males); 16 third-grade children served as reading-matched controls (RC; mean age = 8.80, range: 8.06–10.02; 9 males).

Children within the DD group were first referred by teachers, who were asked to recommend children who performed at the bottom 10% of the class in reading. After parental consent was obtained, children completed assessments of character naming, reading fluency, and non-verbal-IQ using Raven (Raven et al., 2000). The character naming and reading fluency assessments were norm-referenced tests (Xue et al., 2013; Song et al., 2015). The character naming test consists of 150 characters with increasing difficulty. Each child was asked to name characters without the presence of a time constraint. The character naming test has been widely used as an indicator of Chinese literacy skills in children (McBride-Chang et al., 2003; Lei et al., 2011). The reading fluency test required children to silently read up to 100 sentences within 3 min. After reading each sentence, the child was asked to evaluate whether each sentence was literally correct or not. The reliability of the character-naming test is 0.96 and the reliability of the reading fluency test is 0.97 (Xue et al., 2013). The inclusion criteria for the DD group was: (1) a standard score greater than 80 on Raven, and (2) the standard score on either the character naming test or the reading fluency test had to be one standard deviation below the mean.

Children within the AC and RC groups were recommended by teachers based on normal reading achievement. After parental consent was obtained, each child was tested on the Chinese character-naming test. Table 1 lists the raw score on the character-naming test for each of the three groups, along with the *Z*-score for AC and DD based on the age-matched norm (reported in Song et al., 2015). No age-matched norms are available, therefore, we were unable to calculate a *Z*-score for the RC group. An ANOVA was conducted on the raw score for character naming and found a significant group (AC, RC, DD) effect [*F*(2,54) = 39.795, *p* < 0.001]. *Post hoc* *t*-tests revealed that AC had significantly higher scores than DD [*t*(39) = 8.963, *p* < 0.001] and RC [*t*(24) = 4.888, *p* < 0.001], while DD had a significantly greater raw score than RC on the character naming test [*t*(37) = 2.613, *p* = 0.013]. Finally, an ANOVA of group (AC, DD) was conducted on the *z*-score of the character naming test, which revealed a significantly higher score for AC than DD [*F*(1,40) = 85.515, *p* < 0.001].

**Table 1:** Demographic information and Means (standard deviation, range) of the standardized tests and performance on the fMRI task for all three groups of participants.

	RC	AC	DD
*N*	16	19	23
Gender	9 males, 7 females	10 males, 9 females	17 males, 6 females
Age	8.80 (0.66, 8.06–10.02)^∗∗∗^	11.03 (0.46, 10.11–12.03)	11.11 (0.38, 10.11–12)
Non-verbal IQ	–	–	106 (10.94, 81–127)
Reading fluency (raw score)	–	–	49.26 (13.35, 24–78)
Reading fluency (*Z* score)	–	–	–1.10 (0.69, –2.14–0.31)
Character naming (raw score)	98.13 (13.97, 84–134)^∗^	125.39 (6.99, 114–137)^∗∗∗^	107.30 (5.93, 92–119)
Character naming (*Z* score)	–	0.21 (0.58, –0.78–1.16)^∗∗∗^	–1.34 (0.50, –2.63–-0.36)
Accuracy (lexical)	0.92 (0.06, 0.81–1)	0.94 (0.06, 0.70–0.99)	0.91 (0.06, 0.73–0.98)
Accuracy (perceptual)	0.93 (0.13, 0.49–1)	0.95 (0.07, 0.78–1)	0.94 (0.09, 0.67–1)
Reaction time (lexical)	1194 (320, 619–1707)	1294 (333, 620–1906)^∗∗^	1019 (301, 586–1581)
Reaction time (perceptual)	1103 (304, 579–1710)	1242 (322, 544–1861)^∗∗^	948 (351, 499–1595)


*Significant difference with DD (independent-sample t-tests) ^∗^p < 0.05, ^∗∗^p < 0.01, ^∗∗∗^p < 0.001*

An informal interview was conducted with parents to confirm the following inclusionary criteria: (1) native Chinese speaker, (2) right-handed, (3) free of neurological or psychiatric disorders, (4) free of ADHD, Autism, or stuttering, and (5) no metal in the body such as pacemakers, braces, and/or piercings. The Institutional Review Boards at Michigan State University and Beijing Normal University approved the informed consent procedures.

### Lexical and Perceptual Tasks

In the lexical task, two words were presented sequentially in the visual modality, and participants were asked to determine whether the second character of the two words had a similar orthography by sharing a phonetic radical. Each word consisted of two characters. If the word pair shared similar orthography, the participant was asked to press a button on a response pad with the right index finger; if the word pair did not share similar orthography, they were asked to press another button with the right middle finger. Four types of lexical trials were included: similar orthography and phonology (O+P+, e.g., 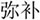/bu3/, 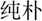/pu3/), similar orthography and different phonology (O+P-, e.g., 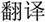/yi4/, 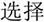/ze2/), different orthography and similar phonology (O-P+, e.g., 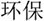/bao3/, 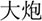/pao4/), and different orthography and phonology (O-P- e.g., 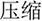/suo1/, 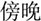/wan3/). The written-word frequency was matched across four conditions (O+P+, 40.5; O+P-, 28.1; O-P+, 27.5; O-P-, 20.2), and was calculated based on the occurrence out of 1 million written words, (Beijing Language and Culture University, 1990). There were 24 word pairs for each trial type. For the perceptual task, two Tibetan symbols were visually presented side-by-side following another two Tibetan symbols. The participant was asked to determine whether the second stimulus matched the first. For example, 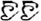 and 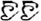 were the same, while 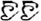 and 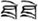 were different. Half of the symbol pairs were same, while the other half were different. There were 24 symbol pairs for the perceptual condition. For the lexical and perceptual tasks, each word/symbol was presented for 800 ms, followed by a 200 ms blank interval. The second word was presented for 800 ms followed by a 2200 to 3400-ms jittered inter-stimulus interval (ISI), during which a red fixation cross (+) would appear on the screen indicating the need to make a response. There were also 48 null trials, which served as resting baseline, in which a black cross changed to red, and participants were asked to press the button with their index finger. Null trials were presented using the same procedure as the lexical and perceptual trials. An event-related design with two 6-min 44 s runs was employed. The presentation order of stimuli in each run was optimized using Optseq^1^.

### MRI Data Acquisition

All MRI images were acquired at Beijing Normal University, Beijing, China, on a 3T Siemens scanner with a standard head coil. MRI scans took place within 2 weeks of standardized testing and practicing of the functional magnetic resonance imaging (fMRI) task. Echo planar imaging (EPI) was used to acquire the BOLD functional images. The following parameters were used: TR = 2000 ms, TE = 20 ms, flip angle = 80°, matrix size = 128 × 128, field of view = 220 mm, slice thickness = 3 mm, number of slices = 33. These scanning parameters resulted in a 1.7 mm × 1.7 mm × 3 mm voxel size. At the beginning of the functional imaging session, T1-weighted structural 3D images were acquired (TR = 2300 ms, TE = 3.29 ms, TI = 900 ms, flip angle = 20°, matrix size = 256 × 256, field of view = 256 mm, slice thickness = 1 mm, number of slices = 160).

### Image Analysis

Data analysis was performed using SPM 12 (Statistical Parametric Mapping)^2^. The images were spatially realigned to the first volume to correct for head movement. Two individuals in the DD group had volumes with more than 3 mm or 3° of movement, however, these volumes counted for less than 10% of the total amount of data for each individual. Artifact Detection Tools (ART) for SPM^3^ was used for head movement correction for trials containing head movement greater than 3 mm. For the AC group and RC group, no participants moved more than 3 mm or 3° during scanning. Functional images were co-registered with the anatomical image and normalized (12 linear affine parameters for brain size and position, 8 non-linear iterations and 2 × 2 × 2 non-linear basis functions) to the standard T1 template volume (MNI). The images were then smoothed with an 8 mm isotropic Gaussian kernel. Statistical analyses at the first level were calculated using an event-related design. A high pass filter with a cutoff period of 128 s was applied. Word pairs were treated as individual events for analysis and modeled using a canonical hemodynamic response function.

A flexible factorial ANOVA of three groups (AC, RC, and DD) by 2 tasks (perceptual and lexical task) was conducted separately on the contrasts lexical minus null and perceptual minus null. All reported results were uncorrected *p* < 0.001 at the voxel level, with voxels > 20, and FDR corrected *p* < 0.05 at the cluster level.

In order to identify the common dyslexia effect shared between the two tasks, two sets of conjunction analyses were conducted. Namely, the conjunction of lexical RC > DD, lexical AC > DD, perceptual RC > DD, and perceptual AC > DD; and the conjunction of lexical DD > RC, lexical DD > AC, perceptual DD > RC, and perceptual DD > AC.

### Psychophysiology Interaction (PPI) Analysis

Psychophysiology interaction was used to calculate functional connectivity in the current study, because it determines which voxels in the brain increase their responses as the influence of a seed region of interest in a given context, such as during a particular behavioral task (O’Reilly et al., 2012). Therefore, it serves our purpose to study different functional connectivity during different tasks.

In the current study, the left MOG and right MOG were selected as seed regions for PPI analysis, as they are two important regions involved in visuo-orthographic processing (Cao et al., 2011). The left precuneus and right pre/post central gyrus were selected as two additional seed regions because of the presence of a dyslexia effect observed during brain activation analysis.

The group peaks for all participants at the four seed regions were identified using an anatomical mask in WFU PickAtlas ^4^, which were the same for the contrast of lexical versus null and perceptual versus null [left middle occipital gyrus (LMOG): *x* = -26, *y* = -92, *z* = 2; RMOG: *x* = 22, *y* = -98, *z* = 2; left precuneus: *x* = -22, *y* = -58, *z* = 34; right pre/postcentral gyrus: *x* = 38, *y* = -22, *z* = 56]. The same group peaks were applied in all three groups because of the similarity of brain activation in these groups at these seed regions. Individualized seed regions were defined as an 8 mm sphere centered at the most significant voxel within 25 mm of the group peak for each individual, with the constraint that the individual’s peak was also within the anatomical mask defined by the WFU PickAtlas. The deconvolved time series of the seed region was extracted for each individual in each task. For the first-level GLM regression analysis, we were interested in finding brain regions where responses were significantly influenced by the interaction of the seed region and the experimental design. In the GLM model, the following regressors were used: the time series of each single seed region, the experimental design, the interaction between the time series of the seed region and the experimental design (lexical versus baseline; perceptual versus baseline), and the six head movement parameters with the interaction between seed region and experimental design as the variable of interest.

An ANOVA of group (RC, AC, and DD) by task (lexical, perceptual) was conducted for each of the four seed regions: left precuneus, right pre/post central gyrus, left MOG, and right MOG in order to investigate how the functional connectivity from each of these seed regions to the whole brain varies in different groups during different tasks. All reported results were uncorrected *p* < 0.001 at the voxel level, with voxels > 20, and FDR corrected *p* < 0.05 at the cluster level.

The ROI analysis was conducted for all connectivity where there was a significant interaction between group and task to examine what was driving the interaction. We created ROIs as spheres, centered at the peak of the interaction effect with a 6 mm radius. For each ROI, group comparisons were conducted using independent sample *t*-tests in SPSS separately for each task.

## Results

### Behavioral Results

**FIGURE 1:**
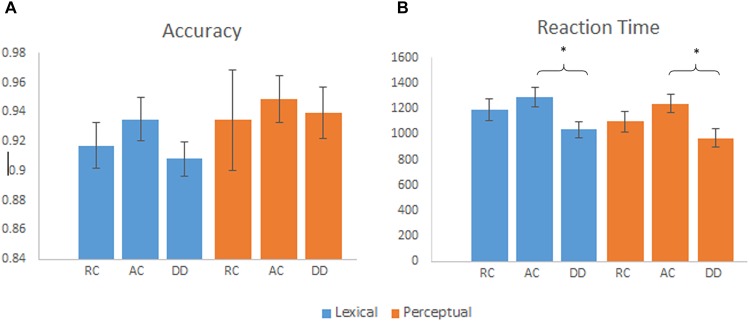
**(A)** Mean accuracy by group for the lexical and perceptual tasks, error bars are standard error. There were no significant group differences on accuracy. **(B)** Mean reaction time by group for the lexical and perceptual tasks, error bars are standard error. DD had a faster reaction time than AC for the lexical and perceptual tasks. ^∗^*p* < 0.01.

We calculated a group (AC, RC, and DD) by task (lexical, perceptual) ANOVA separately for accuracy and RT. It revealed a significant main effect of task [*F*(2,57) = 32.68, *p* < 0.001] for RT, with a faster reaction time for the perceptual task than the lexical task. We also found a significant main effect of group for RT [*F*(2,55) = 4.20, *p* = 0.020]. As indicated in Figure 1, *post hoc* tests revealed that children with DD were faster at responding than the AC group, [*t*(40) = 2.84, *p* = 0.007]. There was no significant difference between DD and RC [*t*(37) = 1.597, *p* = 0.119], or AC and RC [*t*(33) = -1.11, *p* = 0.275]. For accuracy, the main effect of group by task was not significant. There was no group by task interaction for accuracy [*F*(2,55) = 0.21, *p* = 0.811] or RT [*F*(2,55) = 0.71, *p* = 0.499]. For the RT, we ran an ANCOVA of group (AC, RC, and DD) by task (lexical, perceptual) with RT on the null trials as a covariate, we found that the main effect of group was not significant any more [*F*(2,46) = 2.034, *p* = 0.142]. This indicates that the faster RT in the DD group during the lexical and perceptual tasks was not due to a higher performance, but a strategy that they might use by responding fast to everything.

We found a significant effect of lexical condition (O+P+, O+P-, O-P+, O-P-) on accuracy [*F*(3,120) = 5.498, *p* = 0.001], and on reaction time [*F*(3,120) = 9.511, *p* = 0.000], driven by greater accuracy and faster reaction time on the two consistent conditions: O+P+ and O-P- (accuracy: 0.95; RT: 1271) than the two inconsistent conditions: O+P-, O-P+ (Accuracy: 0.91, RT: 1284). It suggests that even though the task can be done perceptually, orthographic and phonological processes are involved.

### Brain Activation Results

The ANOVA of group by task did not show any significant interaction; therefore, we report group differences in each task. In the lexical task, AC had greater activation than DD in the left precuneus and left MOG. RC had greater activation than DD in the left precuneus and bilateral MOG (Table 2). DD showed greater activation than AC in the left STG, right inferior temporal gyrus (ITG), and the right pre/postcentral gyrus (Table 2). DD showed greater activation than RC in the left IFG, and the right pre/postcentral gyrus. RC showed greater activation than AC in the left cingulate gyrus, while AC showed greater activation than RC in the left MTG and right ITG.

**Table 2:** Group comparisons for brain activation in the lexical – null and perceptual – null contrasts, along with the conjunction analysis.

Anatomical region	H	BA	Voxels	*x*	*y*	*z*	*Z*

Lexical-null							
**AC > DD**							
Superior parietal lobule	L	7	162	–20	–68	54	4.62
Superior parietal lobule	R	7	30	28	–66	58	3.97
Postcentral	L	3	54	–32	–26	50	3.80
Middle frontal gyrus	L	6	28	–38	0	38	3.70
Middle frontal gyrus	L	8	25	–46	6	42	3.68
Superior temporal gyrus	L	22	31	–50	–46	10	3.57
**RC > DD**							
Inferior parietal lobule	L	7/40	31	–34	–58	46	3.88
Middle occipital gyrus	L	19	33	–32	–80	–2	3.72
**DD > AC**							
Precentral gyrus	R	4	176	40	–16	54	4.73
Culmen	L	–	32	–14	–52	–20	4.35
Inferior temporal gyrus	R	20	23	52	–10	–24	4.18
Postcentral gyrus	R	3/4	44	52	–14	56	3.95
Superior temporal gyrus	L	38	25	–42	16	–20	3.79
**DD > RC**							
Postcentral gyrus	R	3/4	128	52	–14	52	4.39
Precentral gyrus	R	4	153	40	–18	50	4.27
Postcentral gyrus	L	40	42	–64	–26	22	4.07
Inferior frontal gyrus	L	13	35	–36	12	–14	3.79
Postcentral gyrus	R	2/3	47	64	–22	38	3.71
**AC > RC**							
Cingulate gyrus	L	32	22	–2	16	40	3.90
**RC > AC**							
Middle temporal gyrus	L	21	26	–64	–28	–4	4.04
Inferior temporal gyrus	R	20	23	52	–8	–24	3.80

**Perceptual-null**							

**AC > DD**							
Superior parietal lobule	R	7	85	30	–62	60	4.38
Fusiform gyrus	L	21	43	–18	–74	–16	3.96
Superior temporal gyrus	L	13	23	–58	–42	16	3.93
Precentral gyrus	L	4	82	–32	–24	54	3.90
Lentiform nucleus	L	–	40	–24	–18	6	3.84
Superior parietal lobule	L	7	136	–24	–68	54	3.83
Middle occipital gyrus	R	19	38	26	–66	2	3.75
Precuneus	R	7	30	12	–66	30	3.70
**RC > DD**							
Middle occipital gyrus	L	19	149	–36	–64	–14	4.61
Inferior occipital gyrus	L	19	132	–32	–80	–2	4.25
Precuneus	L	7	93	–18	–58	42	4.03
Cuneus	R	18	22	20	–100	–2	4.00
Precuneus	L	7	38	–14	–72	36	3.98
Precuneus	L	7	32	–26	–70	40	3.66
Superior parietal lobule	L	7	32	–34	–58	50	3.47
**DD > AC**							
*–*	–	–	–	–	–	–	–
**DD > RC**							
Postcentral gyrus	R	3	74	40	–22	50	3.95
**AC > RC**							
–	–	–	–	–	–	–	–
**RC > AC**							
–	–	–	–	–	–	–	–

**Conjunction of lexical and perceptual**							

**AC > DD and RC > DD**							
Precuneus	L	7	34	–16	–62	46	3.02
**DD > AC and DD > RC**							
Precentral gyrus	R	4	20	38	–18	50	3.19


*H, hemisphere; L, left, R, right; BA, Brodmann Area*.

In the perceptual task, AC showed greater activation than DD in the left precuneus, precentral gyrus, lentiform nucleus, right SPL, fusiform gyrus, STG, SPL, MOG, and precuneus. RC showed greater activation than DD in the left MOG, inferior occipital cortex (IOC), precuneus, SPL and right cuneus. DD showed greater activation than AC in the right pre/postcentral gyrus. DD showed greater activation than RC in the right pre/postcentral gyrus. No brain activation differences existed between AC and RC.

No brain regions showed a group by task interaction effect between DD and readers without dyslexia, which included the summation of the AC and RC groups. Detailed brain activation results for each group are reported in the Appendix Table 1.

**FIGURE 2:**
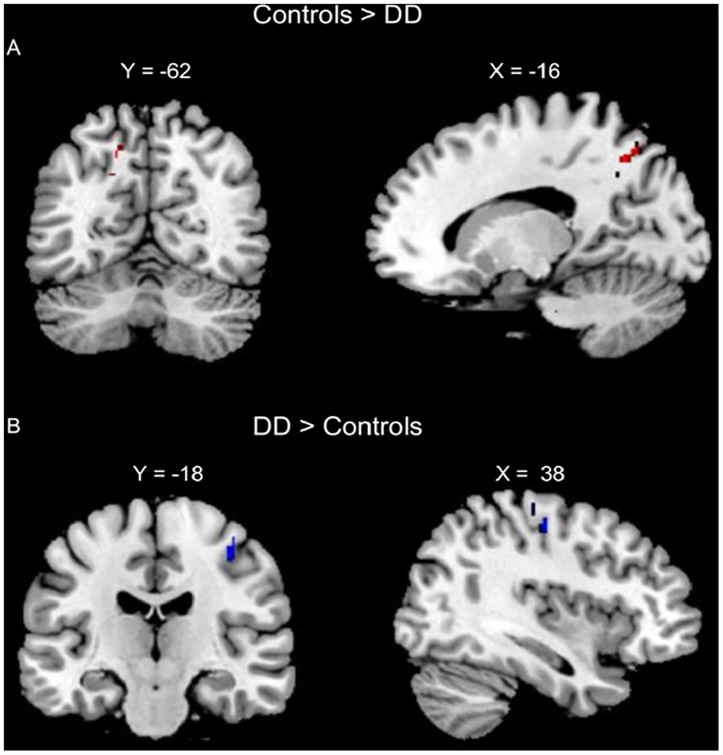
Conjunction analyses of group comparisons in brain activation for the lexical and perceptual tasks. **(A)** Conjunction of AC > DD for the lexical task, RC > DD for the lexical task, AC > DD for the perceptual task, and RC > DD for the perceptual task (left precuneus, in Red). **(B)** Conjunction of DD > AC for the lexical task, DD > RC for the lexical task, DD > AC for the perceptual task, and DD > RC for the perceptual task (right pre/post central gurus, in Blue).

The conjunction analysis of lexical AC > DD, lexical RC > DD, perceptual AC > DD, and perceptual RC > DD revealed an overlap at the left precuneus for the four contrasts. The conjunction analysis of lexical DD > AC, lexical DD > RC, perceptual DD > AC, and perceptual DD > RC showed an overlap at the right pre/postcentral gyrus for the four contrasts, with a conjunction peak at precentral gyrus, which extended to the postcentral gyrus (Table 2 and Figure 2).

### PPI Results

The following reports group by task interaction effect because task specific group differences in the PPI analysis was the focus of the investigation.

**FIGURE 3:**
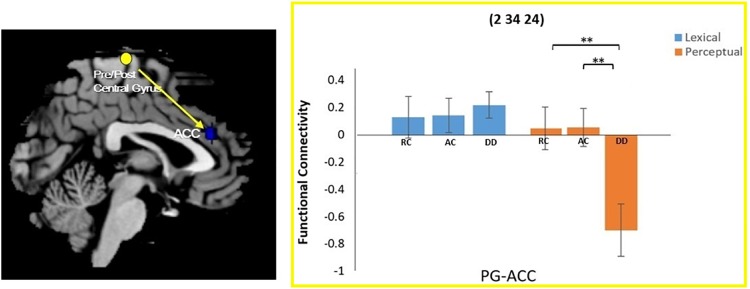
Group × task interaction effect on functional connectivity between the right pre/postcentral gyrus and the right anterior cingulate cortex (ACC). DD had weaker functional connectivity than AC and RC only for the perceptual task. There was no group difference in the lexical task. ^∗∗^*P* < 0.01.

A task by group interaction effect was not observed at the left precuneus seed region. However, an interaction effect for functional connectivity was observed between the right pre/postcentral gyrus seed region and the right anterior cingulate cortex (ACC). Further analysis revealed that the DD group had reduced connectivity between the right pre/postcentral and the ACC compared to AC [*t*(34) = 2.837, *p* = 0.008] and RC [*t*(38) = 3.125, *p* = 0.003] in the perpetual task, but not in the lexical task {DD and AC: [*t*(34) = -0.527, *p* = 0.602]; DD and RC: [*t*(38) = -0.482, *p* = 0.633]} (Figure 3).

**FIGURE 4:**
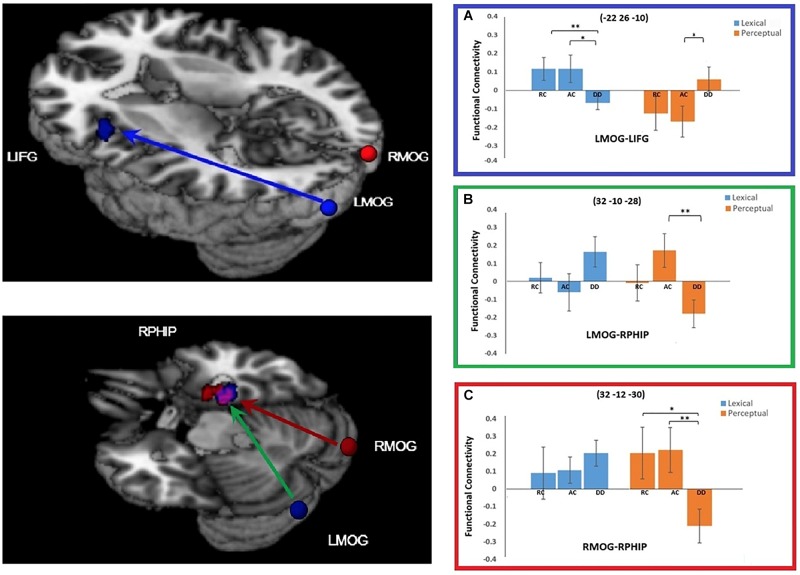
Group × task interaction effects on functional connectivity of the seed regions left middle occipital gyrus (LMOG) and right middle occipital gyrus (RMOG). **(A)** Group × task interaction effect on functional connectivity between the LMOG and the left inferior frontal gyrus (IFG) (in Blue). DD had weaker functional connectivity than AC and RC for the lexical task, and stronger connectivity than AC for the perceptual task. **(B,C)** Group × task interaction effect on functional connectivity between the two seed regions (LMOG and RMOG) and the right parahippocampal gyrus (RPHIP), respectively. From LMOG to RPHIP (in Green), DD had weaker functional connectivity than AC but not RC for the perceptual task. There was no group difference for the lexical task. From the RMOG to the RPHIP (in Red), DD had weaker functional connectivity than RC and AC for the perceptual task. There was no group difference for the lexical task. The interaction effect for the LMOG and the RMOG overlapped at the RPHIP (in purple). ^∗^*P* < 0.05, ^∗∗^*P* < 0.01.

For the connectivity with the left MOG seed region, a significant task by group interaction effect was found in two brain regions: the left IFG and the right parahippocampal gyrus (RPHIP). There was reduced functional connectivity between the left MOG and the left IFG in DD compared to AC [*t*(38) = 2.220, *p* = 0.035] and RC [*t*(34) = 2.733, *p* = 0.010)] in the lexical task. However, in the perceptual task, DD showed increased functional connectivity with the left IFG compared to AC [*t*(38) = -2.167, *p* = 0.037], but not RC [*t*(34) = -1.689, *p* = 0.100]. For the functional connectivity between the LMOG and RPHIP, DD was lower than AC [*t*(38) = 2.931, *p* = 0.006], but not RC [*t*(34) = 1.385, *p* = 0.175] for the perceptual task. However, for the lexical task, DD did not show difference from AC [*t*(38) = -1.699, *p* = 0.098] or RC [*t*(34) = -1.184, *p* = 0.244] (Figure 4).

At the right MOG, we found a significant interaction effect between task and group in the RPHIP, which overlapped with the RPHIP cluster found for the seed region of the LMOG. In the lexical task, we did not find any difference between DD and AC [*t*(38) = -0.919, *p* = 0.364], or between DD and RC [*t*(34) = -0.740, *p* = 0.464]. In the perceptual task, the right MOG showed reduced functional connectivity with the RPHIP for the DD group compared to the AC [*t*(38) = 2.751, *p* = 0.009] and RC groups [*t*(34) = 2.483, *p* = 0.018] (Figure 4).

## Discussion

The present study aimed to identify brain mechanisms underlying visual and orthographic processing in children with DD compared to age-matched and reading-matched control groups. For the brain activation analysis, we found less activation in the left precuneus and greater activation in the right pre/postcentral gyrus for the DD group compared to the AC and RC groups in both the lexical and perceptual tasks, suggesting a common mechanism for visual and orthographic deficits. PPI analysis further revealed interaction effects between task and group, suggesting a task-specific deficit. First, the right pre/postcentral gyrus was less connected with the right ACC in the perceptual, but not the lexical task, for children with DD compared to both control groups. Second, the LMOG was less connected with the left IFG for children with DD compared to the AC and RC groups in the lexical task; however, the LMOG was more connected with the left IFG in the DD group compared to the AC group on the perceptual task. Lastly, the LMOG and right MOG were less connected with the RPHIP in children with DD compared to the control groups in only the perceptual task. Taken together, the brain activation analysis revealed an overlapping brain mechanism associated with orthographic and visual-perceptual deficits in the DD group. However, the PPI analysis revealed task specific deficits during visual and orthographic processing. For the first time, we identified brain mechanisms that are specific for visual-perceptual deficits, specific for orthographic processing deficits and shared by both visual and orthographic deficits.

### Universal Brain Mechanisms of Visual Deficits and Orthographic Deficits

We found that the left precuneus showed decreased activation in children with DD compared to the AC and RC groups for both the lexical and the perceptual tasks. This suggests a universal deficit in orthographic and visual-perceptual processing in dyslexia, which may be related to a deficit in visual attention (Vidyasagar and Pammer, 2010). A previous study reported decreased activation in the left SPL in children with DD, extending to the left precuneus in a complex visual spatial task (Peyrin et al., 2011). The difference found between DD and control groups in the previous study was in the left SPL/precuneus (-15, -56, 48), which is proximal to the peak in the precuneus (-16, -62, 46) in the current study. Therefore, reduced activation in the left precuneus might implicate deficient visual attention that is important in both visual symbolic and orthographic processing. This finding lends support to that of Boros et al. (2016), which demonstrated a similar neural deficit in a letter detection and a symbol string detection task in children with DD. Boros et al. (2016) reported task-universal underactivation in the left visual word-form area of the ventral pathway in children with DD compared to controls. In contrast, we found a universal underactivation in the left precuneus within the dorsal visual pathway in DD across both the lexical and perceptual tasks. The orthographic task in the study conducted by Boros et al. (2016) required participants to identify a single letter in a string of five letters, while the perceptual task required the subjects to detect a single symbol from a string of five symbols. The different tasks and stimuli in the two studies may explain the differences in spatial locations. Our task required the participant to make a same/different judgment on Chinese characters and Tibetan symbols, which may involve a more holistic visual processing, while their detection task likely required a more fine-grained analytic visual processing. However, both studies suggest a universal deficit in linguistic and non-linguistic visual processing. Therefore, even though the current study was conducted in Chinese speaking children, the finding of universal deficits underlying visual perceptual and orthographic processing should not be specific to Chinese readers.

Alternatively, our finding is consistent with previous magnocellular studies, which show reduced brain activation in the left precuneus during visual motion detection and other magnocellular tasks (Peyrin et al., 2011; Reilhac et al., 2013). In summary, we found a universal deficit in linguistic and non-linguistic visual stimuli processing in children with DD, which may be due to deficits in visual attention, or related to magnocellular function abnormality.

Finally, we found an overlap in the increased brain activation at the right pre/postcentral gyrus in the DD group compared to the AC and RC groups in both tasks. A previous study found that children with DD have greater activation of the right pre/postcentral gyrus during an auditory rhyming task than AC and RC groups (Cao et al., 2017). The group difference peak in that task (20, 40, -20) was proximal to the group difference peak in the current study (20, 38, -18). Cao et al. (2017) posited that the enhanced activation of the right pre/postcentral gyrus indicated neural compensation due to an increased reliance on language articulation during auditory rhyming judgment in the DD group. The current study extends previous findings and suggests that the observed neural compensation exists even in non-linguistic tasks. This is consistent with evidence from previous meta-analysis studies which found increased activation of the right pre/postcentral gyrus in those with DD for language, symbol, and number processing (Paulesu et al., 2001; Richlan et al., 2009, 2011). With this in mind, it is possible that children with DD tend to rely on articulation, even for non-linguistic stimuli such as symbols or numbers by saying the name of symbols/shapes, or numbers, which may be to compensate for poor verbal memory.

Moreover, these deficits seem to be associated with dyslexia *per se*, rather than deficient reading because the younger reading-matched control children showed a similar pattern as the older control children. Previous studies with only one AC group cannot exclude the possibility that brain differences observed were actually due to lower reading skill in DD than controls, as intervention studies have found that when reading ability is improved, brain activation patterns become more similar to controls (Simos et al., 2002; Shaywitz et al., 2004). Having two control groups allows researchers to identify brain mechanisms specifically underlying dyslexia instead of low reading ability. However, this does not mean that the brain mechanisms we found are the cause of dyslexia rather than a consequence of dyslexia. As Huettig et al. (2018) argued, the difference between individuals with DD and controls might be a secondary consequence of suboptimal reading experiences including both quantitative and qualitative differences in reading (Huettig et al., 2018). This argument holds true even when children with DD are compared to reading-matched controls, because both the quantity and quality of reading is different in younger reading-matched control children and children with DD. In the current study, children with DD showed faster reaction times than AC and RC, but relatively lower accuracy than the controls. It suggests that children with DD tended to compromise accuracy for faster reaction time, which might be a secondary consequence of suboptimal reading experience. Therefore, the brain differences observed between children with DD and controls might be due to the children with DD using different strategies. In summary, our findings suggest that DD is associated with deficient visual-perceptual processing located at the left precuneus, which might be a cause of dyslexia, or a result from suboptimal reading experience by children with DD.

### Specific Deficits in Visual Perceptual Processing

In the current study, we found that the functional connectivity between the right pre/postcentral gyrus and right ACC was reduced in children with DD compared to the AC and RC groups in the perceptual task, but not in the lexical task. As part of the limbic system, the ACC region is responsible for the complex cognitive operations required for executive control (see a meta-analysis by Margulies et al., 2007), such as bilingual language switching (Abutalebi and Green, 2007) and task-related motor control (Paus et al., 1993). Therefore, the findings of the current study might indicate that the DD group had a possible disassociation between executive control (in the right ACC) and somatosensory/motor processing (in the right PG) during the perceptual task, which may be the underlying mechanism of the visual perceptual deficit. However, in the lexical task, there is an increased demand for executive control due to higher task difficulty than the perceptual trials, and children with DD appear to be capable of maintaining the connection between the right ACC and right pre/postcentral gyrus when the task is harder.

Next, the current study found that the LMOG and right MOG were less connected with the RPHIP in the DD group compared to the AC and RC groups in the perceptual task, but not the lexical task. The parahippocampal gyrus is known to be associated with perceiving the local visual environment in visual navigation (Epstein and Kanwisher, 1998; Park et al., 2011), which processes the layout of local space. It is also involved in processing the semantics of the visual environment (Bonner et al., 2015). The current findings suggest a weaker association between the bilateral visual cortex and the parahippocampal visual network during visual symbol processing in the perceptual task for the children with DD. This further suggests that the visual deficit in DD might be due to the reduced connections between different visual regions.

### Specific Deficit for the Lexical Task

The current study found reduced functional connectivity between the LMOG and the left IFG for the DD group compared to RC and AC groups in the lexical task. In the perceptual task, the DD group showed increased functional connectivity between these two regions compared to AC, but not RC. The LMOG is commonly understood to be responsible for visuo-orthographic processing during written word tasks (Zhang et al., 2013; Cao et al., 2017), while the left IFG plays an important role in phoneme segmentation and manipulation (Booth et al., 2007; Cone et al., 2008). It appears that our finding suggests a reduced interaction between orthography and phonology in DD which is consistent with previous studies (Booth et al., 1999, 2000; Plaut and Booth, 2000; Desroches et al., 2010; Cao et al., 2017). For instance, a behavioral study showed that, compared to typical readers, children with DD had reduced orthographic interference effects (shorter reaction times in responding to orthographically similar words compared to orthographically dissimilar words) in an auditory rhyming task (Zecker, 1991), suggesting less activation of orthography during the phonology task. Subsequently, an fMRI study found that children with DD show less activation in the left fusiform gyrus (a region related to orthographic processing) during auditory rhyming tasks compared to typical readers (Desroches et al., 2010). Weak phonological activation was also found during visuo-orthographic tasks in DD. For example, adults with dyslexia show less activation in the superior temporal gyrus (a region related to phonological processing) during visual word rhyming tasks compared to typical readers (Paulesu et al., 1996). Furthermore, studies have revealed reduced functional connectivity between the left IFG and the left fusiform gyrus in dyslexia during auditory rhyming (Cao et al., 2017), visual rhyming (Cao et al., 2008), phonological-lexical decision (Schurz et al., 2015), visual-lexical decision (van der Mark et al., 2011), and silent reading (Schurz et al., 2015). Our finding is consistent with these findings. Moreover, we further demonstrated that this reduced connectivity between the LMOG and left IFG is only present in the linguistic task. In the perceptual task, children with DD exhibited greater connectivity between LMOG and left IFG than the AC group, which suggests that this connectivity may be more specialized for connecting orthography to phonology in children with typical reading ability while children with DD show a more diffuse pattern across tasks for this connection.

## Conclusion

The present study examined the brain mechanisms involved in visual and orthographic deficits in dyslexia compared to age-matched and reading-matched controls. We found that children with DD had less activation in the left precuneus and greater activation in the right pre/postcentral gyrus compared to AC and RC in both the lexical and perceptual tasks. This suggests a shared mechanism of visual and orthographic deficits in DD. The PPI analysis, however, revealed a task-specific deficit. Children with dyslexia showed reduced connectivity between the LMOG and left IFG in the lexical task, suggesting a weaker connection between orthography and phonology. Furthermore, the children with dyslexia showed reduced connectivity between the bilateral MOG and the right parahippocampal gyrus only in the perceptual task, suggesting a disconnection between different regions in the visual system. In summary, the present study found, both common and specific mechanisms for visual deficits and orthographic deficits in DD, which sheds new light on understanding the visuo-orthographic deficit in developmental dyslexia.

## Ethics Statement

This study was carried out in accordance with the recommendations of the Institutional Review Boards at Michigan State University and Beijing Normal University with written informed consent from all subjects. All subjects gave written informed consent in accordance with the Declaration of Helsinki. The protocol was approved by the Institutional Review Boards at Michigan State University and Beijing Normal University.

## Author Contributions

XY: data collection, data analysis, and paper writing. GS paper writing and editing. YL: PPI analysis. YD: data collection. FC: study design, supervision of study conduction, data analysis, and paper writing.

## Conflict of Interest Statement

The authors declare that the research was conducted in the absence of any commercial or financial relationships that could be construed as a potential conflict of interest.

## Footnotes

1 http://surfer.nmr.mgh.harvard.edu/optseq

2 http://www.fil.ion.ucl.ac.uk/spm

3 https://www.nitrc.org/projects/artifact_detect/

4 fmri.wfubmc.edu/software/pickatlas
