# Supplementary material for: Brain Mechanisms Underlying Visuo-Orthographic Deficits in Children With Developmental Dyslexia
Source: Front Hum Neurosci. 2018 Dec 6;12:490. doi: 10.3389/fnhum.2018.00490 (PMC6291466; doi:10.3389/fnhum.2018.00490)
Supplement: Supplementary file 1 [file Table_1.DOCX]

Appendex1 Table1 Brain activation for three groups of children in the lexical – null and perceptual – null contrasts.

| Anatomical Region | H | BA | Voxels | x | y | z | Z |  |
| --- | --- | --- | --- | --- | --- | --- | --- | --- |
| **LEXICAL-NULL** | | | | | | | | |
| ***AC*** |  |  |  |  |  |  |  |  |
| Inferior frontal gyrus | L | 9 | 19750 | -40 | 4 | 28 | **Inf** |  |
| Superior frontal gyrus | L | 8 | 1370 | -2 | 14 | 54 | **Inf** |  |
| Inferior frontal gyrus | R | 47 | 312 | 30 | 30 | -2 | 5.51 |  |
| Thalamus | L | - | 139 | -10 | -16 | 6 | 4.62 |  |
| Parahippocampal gyrus | R | 36 | 62 | 24 | -26 | -6 | 4.4 |  |
| Lentiform nucleus | L | 51 | 20 | -20 | -10 | -8 | 4.26 |  |
| Caudate body | L | 48 | 34 | -14 | -2 | 22 | 4.03 |  |
| Middle frontal gyrus | R | 6 | 56 | 28 | 2 | 58 | 4 |  |
| Caudate head | R | 48 | 23 | 10 | 18 | 2 | 3.99 |  |
| Superior temporal gyrus | L | 22 | 33 | -48 | 12 | -10 | 3.92 |  |
| Middle frontal gyrus | R | 9 | 27 | 48 | 12 | 30 | 3.88 |  |
| Superior temporal gyrus | L | 22 | 33 | -48 | 12 | -10 | 3.92 |  |
| Middle frontal gyrus | R | 9 | 27 | 48 | 12 | 30 | 3.88 |  |
| Superior temporal gyrus | L | 39 | 55 | -52 | -44 | 10 | 3.84 |  |
| Culmen | R | - | 28 | 22 | -50 | -24 | 3.84 |  |
| Lentiform nucleus | R | 49 | 25 | 22 | 16 | -4 | 3.75 |  |
| **RC** |  |  |  |  |  |  |  |  |
| Cuneus | R | 18 | 5039 | 20 | -98 | 0 | **Inf** |  |
| Inferior occipital gyrus | L | 18 | 5710 | -40 | -84 | -14 | **Inf** |  |
| Inferior frontal gyrus | L | 44 | 1996 | -40 | 10 | 26 | 6.86 |  |
| Parahippocampal gyrus | L | 36 | 102 | -16 | -32 | -4 | 5.83 |  |
| Postcentral gyrus | L | 3 | 633 | -54 | -14 | 50 | 5.76 |  |
| Superior frontal gyrus | L | 8 | 405 | -4 | 14 | 54 | 5.73 |  |
| Middle frontal gyrus | L | 6 | 230 | -32 | -4 | 50 | 5.57 |  |
| Lingual gyrus | L | 18 | 352 | -12 | -60 | 2 | 4.94 |  |
| Parahippocampal gyrus | R | 54 | 37 | 38 | -16 | -20 | 4.7 |  |
| Parahippocampal gyrus | L | 54 | 61 | -34 | -12 | -26 | 4.54 |  |
| Middle temporal gyrus | L | 21 | 52 | -50 | -36 | 4 | 4.53 |  |
| Posterior cingulate | R | 17 | 139 | 14 | -68 | 8 | 4.2 |  |
| Middle temporal gyrus | L | 21 | 25 | -64 | -28 | -2 | 4.17 |  |
| Lentiform nucleus | L | 49 | 29 | -18 | 6 | 0 | 3.78 |  |
| Inferior frontal gyrus | R | 47 | 27 | 30 | 30 | -2 | 3.65 |  |
| ***DD*** |  |  |  |  |  |  |  |  |
| Middle occipital gyrus | R | 18 | 11308 | 22 | -96 | 4 | **Inf** |  |
| Precuneus | L | 7 | 593 | -24 | -60 | 44 | 6.47 |  |
| Inferior frontal gyrus | L | 6 | 1241 | -44 | 8 | 30 | 6.15 |  |
| Middle frontal gyrus | L | 8 | 1036 | -6 | 20 | 48 | 5.54 |  |
| Inferior frontal gyrus | L | 13 | 230 | -28 | 28 | 0 | 5.37 |  |
| Parahippocampal gyrus | R | 27 | 89 | 24 | -28 | -4 | 5.35 |  |
| Thalamus | L | 50 | 229 | -18 | -30 | -2 | 5.22 |  |
| Insula | R | 13 | 297 | 30 | 22 | 8 | 4.87 |  |
| Superior parietal lobule | R | 7 | 175 | 28 | -58 | 44 | 4.85 |  |
| Middle frontal gyrus | L | 6 | 103 | -22 | -12 | 58 | 4.66 |  |
| Anterior cingulate | L | 24 | 94 | -2 | 10 | 26 | 4.47 |  |
| Postcentral gyrus | L | 1 | 131 | -34 | -32 | 48 | 4.32 |  |
| Inferior frontal gyrus | L | 47 | 162 | -42 | 26 | -14 | 4.27 |  |
| Middle frontal gyrus | R | 6 | 80 | 30 | 0 | 54 | 4.13 |  |
| Thalamus | L | 50 | 28 | -14 | -22 | 10 | 4.13 |  |
| Postcentral gyrus | L | 1 | 63 | -48 | -22 | 52 | 4.02 |  |
| Parahippocampal gyrus | R | 27 | 24 | 12 | -38 | -2 | 3.78 |  |
| **Perceptual-null** | | | | | | | | |
| ***AC*** |  |  |  |  |  |  |  |  |
| Cuneus | R | 18 | 7228 | 20 | -98 | 0 | **Inf** |  |
| Inferior occipital gyrus | L | 19 | 6104 | -40 | -84 | -12 | **Inf** |  |
| Thalamus | R | 50 | 99 | 20 | -32 | 0 | 5.66 |  |
| Inferior frontal gyrus | R | 46 | 290 | 42 | 34 | 14 | 5.47 |  |
| Inferior frontal gyrus | R | 44 | 418 | 46 | 8 | 28 | 5.32 |  |
| Precentral gyrus | L | 4 | 603 | -38 | -20 | 48 | 5.1 |  |
| Parahippocampal gyrus | L | - | 39 | -16 | -32 | -4 | 4.79 |  |
| Medial frontal gyrus | R | 6 | 130 | 6 | 26 | 42 | 4.55 |  |
| Middle frontal gyrus | L | 10 | 29 | -48 | 48 | -8 | 4.48 |  |
| Medial frontal gyrus | R | 8 | 56 | 6 | 42 | 46 | 4.48 |  |
| Middle frontal gyrus | R | 47 | 53 | 40 | 44 | -14 | 4.38 |  |
| Parahippocampal gyrus | L | 54 | 53 | -32 | -14 | -24 | 4.09 |  |
| Inferior parietal lobule | R | 40 | 60 | 52 | -36 | 46 | 4.05 |  |
| Cerebellum | R | - | 21 | 18 | -46 | -20 | 3.96 |  |
| Cingulate gyrus | L | 24 | 28 | -2 | 4 | 28 | 3.87 |  |
| Uncus | R | 28 | 25 | 22 | 6 | -28 | 3.86 |  |
| Cerebellum | R | - | 22 | 8 | -74 | -22 | 3.75 |  |
| Inferior frontal gyrus | L | 9 | 28 | -56 | 14 | 30 | 3.73 |  |
| Inferior frontal gyrus | L | 46 | 39 | -46 | 40 | 6 | 3.7 |  |
| Inferior frontal gyrus | L | 44 | 54 | -40 | 8 | 24 | 3.67 |  |
| Inferior frontal gyrus | L | 46 | 20 | -48 | 30 | 20 | 3.48 |  |
| Middle frontal gyrus | L | 6 | 20 | -34 | -4 | 50 | 3.43 |  |
| ***RC*** |  |  |  |  |  |  |  |  |
| Cuneus | R | 18 | 7228 | 20 | -98 | 0 | **Inf** |  |
| Inferior occipital gyrus | L | 19 | 6104 | -40 | -84 | -12 | **Inf** |  |
| Thalamus | R | 50 | 99 | 20 | -32 | 0 | 5.66 |  |
| Inferior frontal gyrus | R | 46 | 290 | 42 | 34 | 14 | 5.47 |  |
| Inferior frontal gyrus | R | 44 | 418 | 46 | 8 | 28 | 5.32 |  |
| Precentral gyrus | L | 4 | 603 | -38 | -20 | 48 | 5.1 |  |
| Parahippocampal gyrus | L |  | 39 | -16 | -32 | -4 | 4.79 |  |
| Medial frontal gyrus | R | 6 | 130 | 6 | 26 | 42 | 4.55 |  |
| Middle frontal gyrus | L | 10 | 29 | -48 | 48 | -8 | 4.48 |  |
| Medial frontal gyrus | R | 8 | 56 | 6 | 42 | 46 | 4.48 |  |
| Middle frontal gyrus | R | 47 | 53 | 40 | 44 | -14 | 4.38 |  |
| Parahippocampal gyrus | L | 54 | 53 | -32 | -14 | -24 | 4.09 |  |
| Inferior parietal lobule | R | 40 | 60 | 52 | -36 | 46 | 4.05 |  |
| Cerebellum | R | - | 21 | 18 | -46 | -20 | 3.96 |  |
| Cingulate gyrus | L | 24 | 28 | -2 | 4 | 28 | 3.87 |  |
| Uncus | R | 28 | 25 | 22 | 6 | -28 | 3.86 |  |
| Cerebellum | R | - | 22 | 8 | -74 | -22 | 3.75 |  |
| Inferior frontal gyrus | L | 9 | 28 | -56 | 14 | 30 | 3.73 |  |
| Inferior frontal gyrus | L | 46 | 39 | -46 | 40 | 6 | 3.7 |  |
| Inferior frontal gyrus | L | 44 | 54 | -40 | 8 | 24 | 3.67 |  |
| Inferior frontal gyrus | L | 46 | 20 | -48 | 30 | 20 | 3.48 |  |
| Middle frontal gyrus | L | 6 | 20 | -34 | -4 | 50 | 3.43 |  |
| ***DD*** |  |  |  |  |  |  |  |  |
| Middle occipital gyrus | R | 18 | 5292 | 38 | -86 | 0 | **Inf** |  |
| Middle occipital gyrus | L | 18 | 3647 | -26 | -88 | 4 | 7.62 |  |
| Thalamus | R | - | 108 | 24 | -28 | -2 | 4.81 |  |
| Thalamus | L | - | 61 | -22 | -34 | -2 | 4.17 |  |
| Lentiform nucleus | R | - | 38 | 20 | 8 | -12 | 3.89 |  |
| Cingulate gyrus | R | 24 | 24 | 4 | 0 | 28 | 3.79 |  |
| Inferior frontal gyrus | R | 9 | 75 | 54 | 12 | 30 | 3.78 |  |
